# The prognostic influence of tumour-infiltrating lymphocytes in cancer: a systematic review with meta-analysis

**DOI:** 10.1038/bjc.2011.189

**Published:** 2011-05-31

**Authors:** M J M Gooden, G H de Bock, N Leffers, T Daemen, H W Nijman

**Affiliations:** 1 Department of Obstetrics and Gynecology, University Medical Center Groningen, University of Groningen, PO Box 30.001, 9700 RB Groningen, The Netherlands; 2 Department of Epidemiology, University Medical Center Groningen, University of Groningen, PO Box 30.001, 9700 RB Groningen, The Netherlands; 3 Department of Medical Microbiology, Molecular Virology section, University Medical Center Groningen, University of Groningen, PO Box 30.001, 9700 RB Groningen, The Netherlands

**Keywords:** tumour-infiltrating lymphocytes, prognosis, meta-analysis

## Abstract

**Background:**

Tumour-infiltrating lymphocytes (TILs) are often found in tumours, presumably reflecting an immune response against the tumour. We carried out a systematic review and meta-analysis, aiming to establish pooled estimates for survival outcomes based on the presence of TILs in cancer.

**Methods:**

A Pubmed and Embase literature search was designed. Studies were included, in which the prognostic significance of intratumoural CD3+, CD4+, CD8+, and FoxP3+ lymphocytes, as well as ratios between these subsets, were determined in solid tumours.

**Results:**

In pooled analysis, CD3+ TILs had a positive effect on survival with a hazard ratio (HR) of 0.58 (95% confidence interval (CI) 0.43–0.78) for death, as did CD8+ TILs with a HR of 0.71 (95% CI 0.62–0.82). FoxP3+ regulatory TILs were not linked to overall survival, with a HR of 1.19 (95% CI 0.84–1.67). The CD8/FoxP3 ratio produced a more impressive HR (risk of death: HR 0.48, 95% CI 0.34–0.68), but was used in relatively few studies. Sample size and follow-up time seemed to influence study outcomes.

**Conclusion:**

Any future studies should be carefully designed, to prevent overestimating the effect of TILs on prognosis. In this context, ratios between TIL subsets may be more informative.

Tumour infiltrating lymphocytes (TILs) are frequently found in tumours, suggesting that tumours trigger an immune response in the host. This so-called tumour immunogenicity is mediated by tumour antigens. These antigens distinguish the tumour from healthy cells, thereby providing an immunological stimulus (Boon *et al*, 1997).

The concept of ‘cancer immunoediting’ describes how the immune system and tumour cells interact during the course of cancer development. It consists of three distinct phases, termed ‘the three E's’ (Kim *et al*, 2007). Elimination entails the complete obliteration of tumour cells by T lymphocytes. In equilibrium, a population of immune-resistant tumour cells appears. Simultaneously, there is an unremitting immunological pressure on non-resistant tumour cells. This phase can last for years (Kim *et al*, 2007). Finally, during escape, the tumour has developed strategies to evade immune detection or destruction. These may be loss of tumour antigens, secretion of inhibitory cytokines, or downregulation of major histocompatibility complex molecules (Stewart and Abrams, 2008). Additionally, antigens may be ineffectively presented to the immune system, that is, without appropriate co-stimulation, resulting in immunological tolerance (Stewart and Abrams, 2008).

Many studies report a survival benefit associated with the presence of TIL (Zhang *et al*, 2003; Sato *et al*, 2005; Galon *et al*, 2006; Leffers *et al*, 2009). This suggests that TILs are effective at delaying tumour progression, despite being antagonised by the mechanisms mentioned above. However, it is important to distinguish between different types of T lymphocytes, because they all have different functions in the tumour microenvironment.

CD8+ cytotoxic T lymphocytes (CTLs) are directly capable of killing tumour cells. CD4+ T helper lymphocytes (Th) are a heterogeneous cytokine secreting class of T lymphocytes. T helper type 1 lymphocytes (Th1) have a crucial role in activating CTLs. T helper type 2 lymphocytes stimulate humoral immunity and activate eosinophils. In terms of antitumour immunity, Th2 activation is less effective than Th1 activation (Yu and Fu, 2006). Besides the Th1 and Th2 subsets, a CD4+ regulatory T lymphocyte (Treg) subset suppresses effector T lymphocytes (Curiel *et al*, 2004). In cancer, Treg preferentially traffic to tumours, as a result of chemokines produced by tumour cells and microenvironmental macrophages (Curiel *et al*, 2004). In recent years, the hypothesis that ratios between different subsets are most predictive of prognosis has gained much attention. Frequently used ratios are CD8+/FoxP3+ (effector : regulatory) ratio and CD8+/CD4+ (effector : helper) ratio. These measures may provide a more comprehensive view of the events at the site of disease, as the immune system is not a collection of solitary agents, but rather a complex system of checks and balances – each subset being dependent upon collaboration with and authorisation from other subsets.

A commonly used approach to gain more insight in the *in vivo* interaction between tumours and the immune system is to quantify the numbers of TILs, and to relate these to tumour characteristics and prognostic outcome. These studies have been carried out across many types of cancer, and many types of TIL, with widely differing sample sizes. We were interested in obtaining a more precise estimate of the effect of TIL on survival. Therefore, we undertook a systematic review and meta-analysis, aiming to establish pooled estimates for survival outcomes based on the presence of TILs in different types of cancer. We assumed that the direction of prognostic influence of TILs would be the same in all solid tumour types, but that only the magnitude of this effect might differ between tumour locations and/or stage of disease. Therefore, we felt it was justified not to focus on one particular tumour type.

## Methods

### Search strategy

We designed a broad PubMed and Embase search, using the following terms: prognosis[tw], prognos^*^[tw], mortality[tw], surviv^*^[tw], survival[tw], disease free survival, disease specific survival, progression free survival, tumor infiltrating lymphocyte^*^, intratumoral lymphocyte^*^, intratumoural lymphocyte^*^, intra-tumoural lymphocyte^*^, intra-tumoral lymphocyte^*^, TIL[tw], cancer[tw], malignancy[tw], malignan^*^[tw], neoplasm^*^[tw], tumor^*^[tw], tumour^*^[tw], carcinoma^*^[tw]. We used the following MeSH terms: ‘prognosis’, ‘mortality’, ‘survival’, ‘survival analysis’, ‘disease-free survival’, ‘lymphocytes, tumor-infiltrating’, ‘CD4+-Positive T-Lymphocytes’, ‘CD8+-Positive T-Lymphocytes’, ‘neoplasms’. Additionally, possible missing papers were searched in reference lists of selected papers and related articles as suggested by PubMed.

### Inclusion criteria

We only included studies, in which the prognostic significance of CD3+, CD4+, CD8+, and FoxP3+ lymphocytes was examined, including ratios between these subsets. These lymphocyte markers were chosen based on the assumption that these were the most frequently used markers. All papers in which only haematoxylin and eosin stained slides were used, or which did not incorporate a time-to-event survival analysis, were excluded. Similarly, immunological clinical trials were rejected, because active immunotherapy aims to modify the presence or the composition of T-lymphocyte subsets. We, however, were only interested in the prognostic relevance of TILs in the naturally occurring immunological situation. Furthermore, we also excluded *in vitro* and animal studies.

Only studies regarding intratumoural lymphocytes were included. The analysis of lymphocytes in tumour stroma was an exclusion criterion. This also applied to stromal lymphocytes combined with intratumoural lymphocytes (e.g., ‘tumour and surrounding stroma’). To be sure that the same definition of ‘intratumoural’ was used in all included papers, we excluded all studies in that the lymphocyte location was not clearly specified.

We included studies in solid tumours of any kind. Haematological malignancies were excluded, because these are malignancies of the immune cells themselves.

To increase the power of our analysis, it was decided to only include larger studies with *n*⩾100 patients, to avoid publication bias that might exist among small studies.

Finally, all included papers had to be published between January 2003 and February 2011 and written in English. In early 2003, the landmark paper by Zhang *et al* was published (Zhang *et al*, 2003). This paper subsequently inspired many authors to determine the prognostic significance of TILs in many types of cancer, and thereby formed a rational starting point for our literature search.

**Figure 1:**
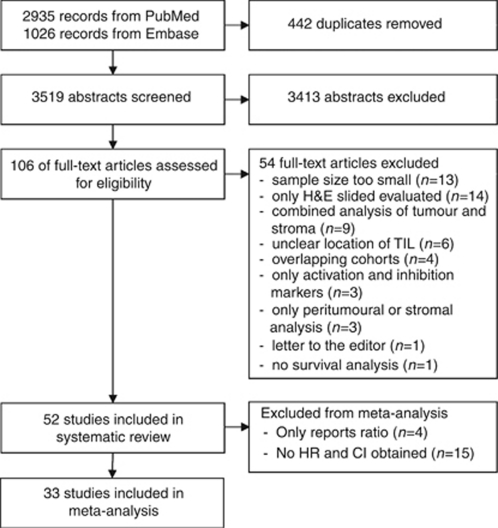
Flowchart of study selection process.

Figure 1 shows a flowchart of the study selection process. The search yielded 2935 papers in Pubmed and 1026 papers in Embase, 584 of which were not found in Pubmed. Thus, 3519 unique papers were found. With the inclusion criteria mentioned above, the full-text version of 106 papers was reviewed. Of these papers, another 54 were excluded because they did not fit the inclusion criteria. Specifically, four of these 54 were excluded because of the use of the same cohorts. Zlobec *et al* (2007, 2008a, 2008b), Lugli *et al* (2009), and Baker *et al* (2007) all used (selections of) the same cohort. One paper by Zlobec *et al* (2007) was selected based on the reporting of hazard ratios and the use of the largest cohort. Papers by Milne *et al* (2009) and Clarke *et al* (2009) also stem from the same tissue microarray. As Milne *et al* did not report hazard ratios, this paper was excluded, except in case of the FoxP3+ staining, which was not reported by Clarke *et al.* Finally, the cohorts used by Galon *et al* (2006) and Pages *et al* (2009) were the same. Pages *et al* paper was excluded, as only Galon *et al* paper enabled the estimation of hazard ratios (HRs) (see: Statistical analysis). Thus, 52 papers were included in the systematic review part of the study.

### Data extraction

Data were extracted using a predefined form, recording: author, journal, year of publication, tumour type, lymphocyte subsets, location of lymphocytes, median follow-up time, scoring methods, cut-offs for positive expression, number of TILs-low and TILs-high patients, outcome of univariate and/or multivariate analysis (including *P*-values, hazard ratios, and 95% confidence intervals) and other major study outcomes.

Unless indicated, we only report outcomes from the whole cohort included in these studies. Subgroup analyses are not specifically mentioned. Some studies were entirely carried out in a subgroup of patients, for instance only in advanced stage patients. These studies are specified in the Supplementary Tables.

The results from univariate Cox regression, that is, HRs and 95% confidence intervals (CIs), were used for meta-analysis. For all lymphocyte ratios other than CD8/FoxP3, there were not enough studies available to carry out meta-analysis. Therefore, all studies that only reported only these lymphocyte ratios, and not the results from the lymphocyte subsets on their own, were excluded from meta-analysis (Hiraoka *et al*, 2006b; Kobayashi *et al*, 2007; Han *et al*, 2008).

Thus, we attempted to carry out meta-analysis on 49 studies, which requires HRs and 95% CIs from univariate Cox regression analysis. Nine of these 49 papers reported HRs and 95% CIs for all stainings (Sato *et al*, 2005; Gao *et al*, 2007; Nedergaard *et al*, 2007; Siddiqui *et al*, 2007; Zlobec *et al*, 2007; Lee *et al*, 2008; Li *et al*, 2009; Shen *et al*, 2010; Zingg *et al*, 2010). Three papers only reported some stainings (Jordanova *et al*, 2008; Clarke *et al*, 2009; Sinicrope *et al*, 2009), and in two papers only stratified or subgroup analyses were mentioned (Nosho *et al*, 2010; de Kruijf *et al*, 2010). In all, 35 papers did not report any HRs and CIs for progression or survival (Cho *et al*, 2003; Toomey *et al*, 2003; Wakabayashi *et al*, 2003; Zhang *et al*, 2003; Chiba *et al*, 2004; Prall *et al*, 2004; Baeten *et al*, 2006; Bates *et al*, 2006; Cai *et al*, 2006, 2009; Galon *et al*, 2006; Hiraoka *et al*, 2006a; Al Shibli *et al*, 2008; Callahan *et al*, 2008; Heimberger *et al*, 2008; Kawai *et al*, 2008; Perrone *et al*, 2008; Sasaki *et al*, 2008; Shah *et al*, 2008; Tomsova *et al*, 2008; Adams *et al*, 2009; De Jong *et al*, 2009; Gobert *et al*, 2009; Jensen *et al*, 2009; Leffers *et al*, 2009; Milne *et al*, 2009; Ruffini *et al*, 2009; Stumpf *et al*, 2009; Al Attar *et al*, 2010; Barnett *et al*, 2010; Deschoolmeester *et al*, 2010; Kasajima *et al*, 2010; Shimizu *et al*, 2010; Simpson *et al*, 2010; Sorbye *et al*, 2011). The authors of 40 papers with (partially) missing data were contacted, but we were unable to trace three authors (Cho *et al*, 2003; Wakabayashi *et al*, 2003; Baeten *et al*, 2006). In all, 15 authors very kindly sent us the requested data (Chiba *et al*, 2004; Prall *et al*, 2004; Bates *et al*, 2006; Hiraoka *et al*, 2006a; Jordanova *et al*, 2008; Sasaki *et al*, 2008; Cai *et al*, 2009; Clarke *et al*, 2009; De Jong *et al*, 2009; Jensen *et al*, 2009; Leffers *et al*, 2009; Milne *et al*, 2009; Ruffini *et al*, 2009; Barnett *et al*, 2010; Sorbye *et al*, 2011). For the remaining studies, estimations of HRs and 95% CIs were attempted using the spreadsheet provided by Tierney *et al* (2007. This was successful in eight cases (Zhang *et al*, 2003; Galon *et al*, 2006; Al Shibli *et al*, 2008; Perrone *et al*, 2008; Tomsova *et al*, 2008; Adams *et al*, 2009; Sinicrope *et al*, 2009; Kasajima *et al*, 2010). In cases with partially missing data, the available data were used (de Kruijf *et al*, 2010; Nosho *et al*, 2010). Finally, fifteen studies for that no HRs and CIs could be obtained, were excluded from meta-analysis and only included in the systematic review part of this paper (Cho *et al*, 2003; Toomey *et al*, 2003; Wakabayashi *et al*, 2003; Baeten *et al*, 2006; Cai *et al*, 2006; Callahan *et al*, 2008; Heimberger *et al*, 2008; Kawai *et al*, 2008; Shah *et al*, 2008; Gobert *et al*, 2009; Stumpf *et al*, 2009; Al Attar *et al*, 2010; Deschoolmeester *et al*, 2010; Shimizu *et al*, 2010; Simpson *et al*, 2010). Thus, ultimately 34 out of 49 studies were included in the meta-analysis part of this study.

### Assessment of study quality

Study quality was assessed using the predefined form by De Graeff (de Graeff *et al*, 2009), which was adapted from Hayes (Hayes *et al*, 1996) and McShane (McShane *et al*, 2005). Briefly, the following criteria were scored: (1) Are in- and exclusion criteria defined? (2) Is the study prospective or retrospective? (3) Are the clinical and pathological characteristics of the patients sufficiently described? (4) Is the method used sufficiently described? (5) Is the outcome measure defined? (6) Is the follow-up time recorded? (7) Does the study report the number of patients lost to follow-up or otherwise unavailable for statistical analysis? (Supplementary Table 1). As this quality score is not validated, we did not exclude studies based on a low score.

### Statistical analysis

All calculations were performed with HRs defined as the risk of death or progression for high TILs *vs* low TILs tumours. In studies that reported HRs for low TILs *vs* high TILs, the reciprocal of the HRs and CIs was taken to calculate the results the other way around.

Meta-analysis is generally carried out with the natural logarithm of the HR and its standard error, to make the range of HRs symmetrical. After log transformation, a HR of 0 becomes minus infinity, a HR of 1 becomes 0, and a HR of infinity remains infinity (Higgings and Green, 2009). We calculated the log hazard ratio and associated standard errors using the spreadsheet provided by Tierney *et al* (2007).

Next, meta-analysis was carried out with the DerSimonian Laird model for random effects, using the inverse of variance as a weighing factor. All analyses were stratified by T-lymphocyte subset. The *I*^2^ statistic was used to evaluate heterogeneity. A value >50% on the scale of 0–100% was considered to indicate substantial heterogeneity between studies. Funnel plots were constructed to assess publication and/or selection bias.

The studies in this systematic review and meta-analysis vary widely with regard to methodology. We wondered whether these differences would affect study outcomes. Additionally, we wanted to identify sources of heterogeneity, which appeared in pooled analyses. Therefore, stratified analyses were carried out for CD3+ and CD8+, because these subsets were investigated in the largest number of studies. All stratified analyses were carried out with overall survival only to increase uniformity. Differences between strata were assessed using the test for subgroup differences in Review Manager (The Cochrane Collaboration, The Nordic Cochrane Centre, Copenhagen, Denmark).

All analyses were carried out using SPSS version 16.0 (SPSS, Chicago, IL, USA) and Review Manager version 5.0.

## Results

### Study characteristics

The 52 studies had a median quality score of 5 out of 8 (range: 3–8) and consisted of a median of 160 patients (range: 100–1290), with a median follow-up of 47 months (range: 20–228), and were published in journals with a median impact factor of 5.07 (range: 1.15–47.05). All studies used immunohistochemistry as method for detecting TILs.

Table 1 summarises some important study characteristics. Most studies were carried out in ovarian cancer, but studies in colorectal cancer (CRC) contained most patients. CD8+ was by far the most popular lymphocyte marker, as it was quantified in 73.3% of patients. Most studies used whole-tissue slides to evaluate TILs, but studies in which tissue microarrays (TMAs) were used, included more patients. This is consistent with the fact that TMAs are especially suited for high-throughput analysis. Finally, counting TILs in representative areas of tumour was more popular than specifically selecting hotspots with highest infiltration rates.

### Pooled analysis

**Figure 2:**
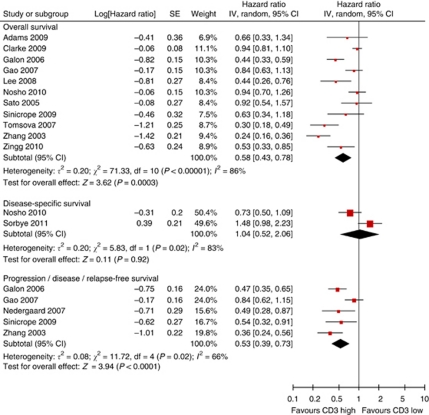
Forest plots of studies on CD3+ TILs. Hazard ratios and 95% confidence intervals from individual studies are depicted as squares and horizontal lines, respectively. The pooled estimate is shown as a diamond shape, where the center represents the pooled HR and the horizontal borders represent the 95% CI. Hazard ratios are defined as high CD3 *vs* low CD3 counts, therefore a hazard ratio <1 represents a lower risk of death or progression associated with high CD3 counts.

We carried out meta-analysis under the assumption of homogeneity, stratified by T-lymphocyte subset. For CD3+, a general T-lymphocyte marker, the results are shown in Figure 2. The pooled HR and CI for overall and progression-free survival are very similar, both pointing to a survival advantage associated with presence of TILs (HR 0.58, 95% CI of 0.43–0.78 for death, HR 0.53, 95% CI 0.39–0.73 for progression). Only two studies used disease-specific survival, with opposing results. However, there is a considerable degree of heterogeneity in the analyses, as well as slight asymmetry in the funnel plots (Figure 5A). Eight studies could not be included in meta-analysis, because no HRs and CIs were reported (Supplementary Table 2, Toomey *et al*, 2003; Baeten *et al*, 2006; Cai *et al*, 2006; Shah *et al*, 2008; Stumpf *et al*, 2009; Al Attar *et al*, 2010; Deschoolmeester *et al*, 2010; Simpson *et al*, 2010). Three studies found significant positive influence of CD3+ TILs on overall survival (Al Attar *et al*, 2010), disease-specific survival (Simpson *et al*, 2010), and disease-free survival (Cai *et al*, 2006), but four found no effect on overall survival (Toomey *et al*, 2003; Baeten *et al*, 2006; Shah *et al*, 2008; Stumpf *et al*, 2009). Of these four, two did find that progression-free or disease-free survival improved with CD3+ infiltration (Toomey *et al*, 2003; Stumpf *et al*, 2009). The remaining study describes beneficial effects of CD3+ TILs on disease-free survival, but not on overall survival (Deschoolmeester *et al*, 2010).

**Figure 3:**
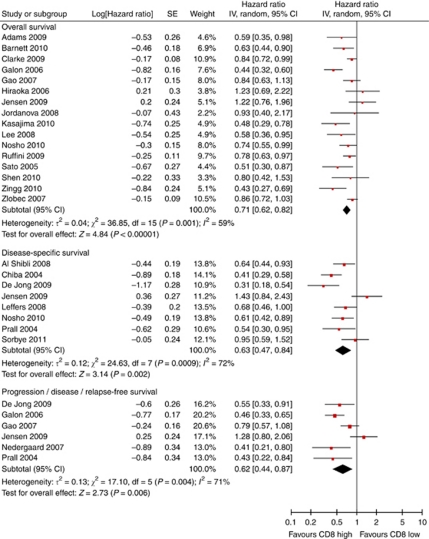
Forest plots of studies on CD8+ TILs. Hazard ratios and 95% confidence intervals for death or progression associated with high *vs* low CD8 counts.

For CD8+, 23 studies were included in meta-analysis. The presence of CD8+ results in prognostic advantages for all survival endpoints tested (Figure 3). Again, there was a considerable amount of heterogeneity present, but the funnel plot was more symmetric (Figure 5B). Of the eight studies that could not be included in meta-analysis, seven reported improved overall survival (Cho *et al*, 2003; Baeten *et al*, 2006; Cai *et al*, 2006; Callahan *et al*, 2008; Kawai *et al*, 2008; Stumpf *et al*, 2009; Deschoolmeester *et al*, 2010). One study found a negative effect of CD8+ TIL on survival, but this did not reach statistical significance in multivariate analysis (Wakabayashi *et al*, 2003) (Supplementary Table 4).

Meta-analysis of the six studies reporting overall survival in CD4+ revealed a pooled HR of 0.82, with a 95% CI of 0.69–0.98, which is statistically significant (*P*=0.03, data not shown). Heterogeneity was 0%. Progression-free survival (Gao *et al*, 2007; Nedergaard *et al*, 2007; Li *et al*, 2009) and disease-specific survival (Al Shibli *et al*, 2008; Sorbye *et al*, 2011) were not influenced by CD4+ TILs in pooled analysis (data not shown). Four papers were excluded from pooled analysis because Cox regression analysis was not performed. One of these report improved overall survival (Cho *et al*, 2003), but three other papers do not find a statistical significant effect of CD4+ TILs (Wakabayashi *et al*, 2003; Jordanova *et al*, 2008; Stumpf *et al*, 2009) (Supplementary Table 3).

**Figure 4:**
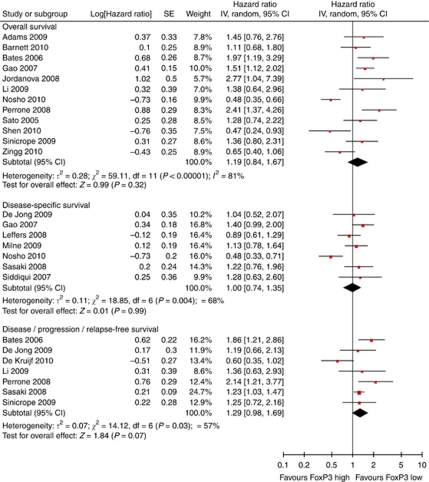
Forest plots of studies on FoxP3+ TILs. Hazard ratios and 95% confidence intervals for death or progression associated with high *vs* low FoxP3 counts.

**Figure 5:**
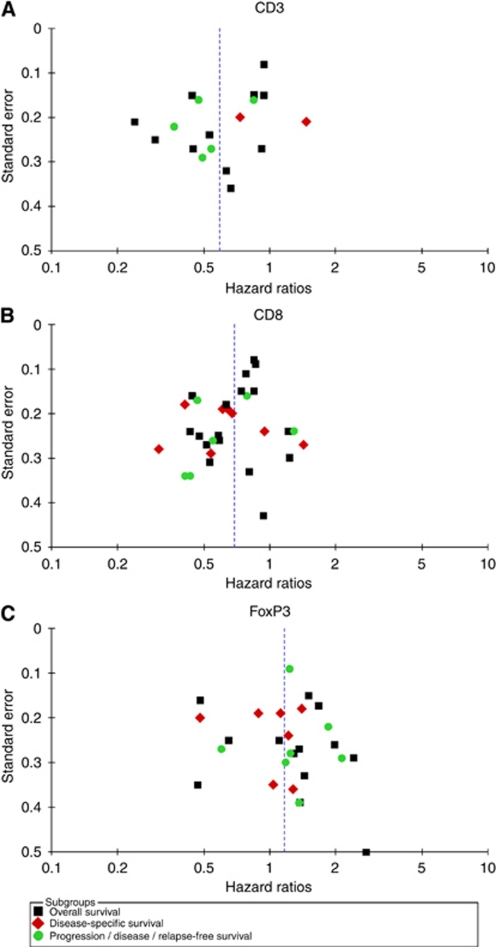
Funnel plots. Funnel plots showing the associations between hazard ratios and s.e. for individual studies. (**A**) CD3+ TILs. (**B**) CD8+ TILs. (**C**) FoxP3+ TILs.

FoxP3+ is a relatively selective Treg marker (Hori *et al*, 2003) and was used in all 22 studies we included. Surprisingly, meta-analysis on 18 of these showed no statistically significant impact on overall, disease-specific, or progression-free survival (Figure 4). Heterogeneity was present, and the funnel plot was slightly asymmetric (Figure 5C). Four studies were excluded from meta-analysis, two of these found no prognostic significance of Treg (Shah *et al*, 2008; Gobert *et al*, 2009), whereas the remaining studies showed that Treg infiltration was associated with improved survival (Heimberger *et al*, 2008) and reduced relapse-free survival (Shimizu *et al*, 2010) (Supplementary Table 5).

Six studies have examined both CD4+ and FoxP3+ (Sato *et al*, 2005; Gao *et al*, 2007; Jordanova *et al*, 2008; Li *et al*, 2009; Shen *et al*, 2010; Zingg *et al*, 2010). Jordanova *et al* (2008) and Gao *et al* (2007) *et al* found a no effect of CD4+, but a negative effect of FoxP3+ in univariate analysis in cervical and hepatocellular cancer, respectively. However, only Jordanova carried out multivariate analysis, in which this effect did not hold. The remaining studies observe a prognostic effect of neither CD4+ nor FoxP3+ in esophageal, gastric, ovarian, and renal cell cancer (Sato *et al*, 2005; Li *et al*, 2009; Shen *et al*, 2010; Zingg *et al*, 2010).

### Stratified analysis

In case of CD3+, the pooled results from the seven smallest studies were strongly significant, whereas this was not the case with four larger studies (Table 2). Similarly, studies with a shorter follow-up and high quality score showed a statistically significant pooled result, whereas this was not the case for those with a long follow-up or a low quality score. However, follow-up duration was not reported in all studies. Interestingly, the choice of tissue type also seemed to be influential. Using whole-tissue slides as opposed to a TMA resulted in a lower pooled HR. Stratifying for tumour type was not entirely feasible, because of insufficient studies in similar cancer types. However, when comparing the two most popular malignancies, there were no significant differences between results in ovarian and colorectal cancer. Overall, heterogeneity was not decreased by performing stratified analysis, and is therefore from thus far unknown origins.

For CD8+, the beneficial prognostic significance of CD8+ infiltration was more pronounced in studies with fewer patients and shorter median follow-up time. Again, ovarian and colorectal cancers were the most frequently used types of cancer, both with similar outcomes. Heterogeneity seemed to be especially affected by study size and follow-up duration, but the latter is probably influenced by the exclusion from this analysis of four studies, which did not report follow up.

### Ratios between T-lymphocyte subsets

Relatively few studies incorporated T-lymphocyte ratios (Supplementary Table 6). Moreover, the use of different survival outcomes (overall, disease-specific, disease-free, and relapse-free survival) decreased the potential for pooled analysis even further. Therefore, pooled analysis was only possible for the CD8/FoxP3 ratio.

Pooled analysis for the six studies reporting overall survival based on CD8/FoxP3 ratios was strongly significant with relatively low heterogeneity (HR 0.48, 95% CI 0.34–0.68, *P*<0.0001, *I*^*2*^=49%) (Sato *et al*, 2005; Jordanova *et al*, 2008; Cai *et al*, 2009; Barnett *et al*, 2010; Shen *et al*, 2010; Zingg *et al*, 2010). Furthermore, two studies report positive effects of a high CD8/FoxP3 ratio on disease-specific (De Jong *et al*, 2009; Leffers *et al*, 2009) and progression-free survival (Cai *et al*, 2009; De Jong *et al*, 2009).

In three studies, the CD3+/CD8+ ratio was used, but each used a different interpretation of this ratio. Han *et al* (2008) found an independent positive effect of either CD3+ or CD8+ compared with no CD3+ or CD8+ in ovarian cancer. In gastric cancer, Lee *et al* (2008) observed that high numbers of both CD3+ and CD8+ are favorable compared with low numbers of both cell types. Finally, Kobayashi *et al* (2007) found that high numbers of CD8+ compared with CD3+ was not a prognostic factor in hepatocellular cancer. Naturally, for this ratio it is important to keep in mind that most CD8+ cytotoxic lymphocytes are also CD3+.

The CD8+/CD4+ ratio was used in three studies (Sato *et al*, 2005; Jordanova *et al*, 2008; Zingg *et al*, 2010), and found to be a positive prognostic predictor in one of these (Sato *et al*, 2005). Importantly, the CD4+ component also contains Treg. Thus, this ratio is more difficult to interpret than the CD8+/FoxP3+ ratio, as the CD4+ population can be very mixed.

The FoxP3+/CD3+ and FoxP3+/CD4+ ratio were used in one (Sinicrope *et al*, 2009) and two (Hiraoka *et al*, 2006b; Kobayashi *et al*, 2007) studies, respectively. All three studies found a negative prognostic effect associated with FoxP3+ preponderance.

## Discussion

Quantifying TILs by histopathology is a frequently used approach to gain insight in the immunological activity against tumours. In this systematic review and meta-analysis, we analysed larger studies of recent years to determine which similarities and differences exist between their results.

CD3+ and CD8+ TILs turned out to have a positive effect on prognosis in meta-analysis, with HRs of 0.58 (95% CI 0.43–0.78) and 0.71 (95% CI 0.62–0.82), respectively, for death from all causes. CD4+ TILs were associated with a slightly improved overall survival (HR 0.82, 95% CI 0.69–0.98), but its FoxP3+ regulatory subset not associated with overall survival (HR 1.19, 95% CI 0.84–1.67). All in all, these HRs represent statistically significant, but not dramatic differences in survival. The CD8/FoxP3 ratio produced a more impressive HR (risk of death: HR 0.48, 95% CI 0.34–0.68) but was used in relatively few studies. These results underline the need to examine FoxP3 and CD8 together.

We carried out this meta-analysis assuming that the prognostic effect of TILs would not differ greatly between types of cancer. We considered it unlikely that TILs infiltration strongly improves prognosis in one type of cancer, but has the complete opposite effect in another. However, clinicopathological factors might affect the impact of TILs on prognosis, that is, HRs moving closer to (but not crossing) 1 in high stage or grade. We attempted to test this hypothesis *post hoc* in stratified analysis. These analyses were limited by the fact that sufficient numbers of studies were only available for ovarian and colorectal cancer. For these tumour types, we found no significant differences between pooled outcomes. Moreover, heterogeneity was not clearly affected. This indicates that the prognostic effects of CD3+ and CD8+ TILs are similar in ovarian and colorectal cancer and that heterogeneity in pooled analysis was caused by factors other than tumour type.

Furthermore, stratified analysis provided some hints that methodological aspects such as sample size and follow-up time may have influenced outcomes of studies on CD3+ and CD8+ TILs. For CD3+ and CD8+ TILs, smaller studies produced more dramatic HRs than larger studies. Additionally, studies with a longer follow-up time were less likely to produce statistically significant results. Unfortunately, as not all studies report median follow-up times, these results are tentative. Differences in significant results based on sample size or follow up time may be caused by publication bias. This can be detected by funnel plots, but these are relatively crude and have a tendency for false positivity. In our case, the funnel plots should be interpreted with caution as the number of studies in our meta-analysis is relatively small and consist of different populations (Lau *et al*, 2006). This means that the asymmetry may not just be caused by publication or selection bias, but also by inherent differences in study populations (Lau *et al*, 2006). Thus, although some asymmetry seemed to be present, we cannot conclude with certainty whether publication bias was an issue.

In addition to the factors we tested in subgroup analysis, the determination of cutoff points also differed widely. Some studies use percentiles, tertiles or the median, whereas others use absence *vs* presence, the minimal *P*-value approach, or do not report a cutoff point at all. All studies used immunohistochemistry, which is notorious for its variability due to factors related to staining protocols or tissue fixation techniques. It is an attractive technique to use as primary screening, but the next step should be to validate the results in other models.

These results raise the question whether biology or methodology is the source of the observed prognostic effects of TILs on survival. Biological support can be gained from studies into T-lymphocyte kinetics, which offer a more detailed perspective. It has been shown that immune cells can proliferate *in vitro* in response to tumour-specific antigens, and that the influx of immune cells into a tumour results in the induction of an inflammatory microenvironment (reviewed in Kim *et al*, 2007; Finn, 2008). Murine studies also demonstrated the potency of the immune system, when adoptive transfer of tumour-specific CD8+ T-lymphocytes resulted in complete eradication or regression of established tumours (Vierboom *et al*, 1997; Palmer *et al*, 2004; Ruttinger *et al*, 2004; Wall *et al*, 2007). Also, the increase in cancer risk seen after solid organ transplantation, when immunosuppressive drugs are used, suggests a prominent role for immune surveillance (Kim *et al*, 2007).

Nonetheless, several biological mechanisms may at the same time prevent antitumour responses of TILs. For instance, lymphocytes present in the tumour might not always be active, due to immune escape or tolerance. Alternatively, the immune response may be skewed towards relatively ineffective Th2 or Treg responses. Tumour-infiltrating lymphocytes may also be properly activated, but simply out of their league because of the speed of tumour growth. However, these hypotheses cannot be fully investigated in immunohistochemical studies. Some attempts at a more functional perspective have been made by staining for activation markers on lymphocytes such as Granzyme-B (Oshikiri *et al*, 2003; Gao *et al*, 2007), CD25 (Ladanyi *et al*, 2004), OX40 (CD134) (Ladanyi *et al*, 2004) and CD69 (Hillen *et al*, 2008), or inhibiting co-stimulatory molecules such as PD-1 (Thompson *et al*, 2007) and its ligand B7-H1 (Thompson *et al*, 2005; Boorjian *et al*, 2008). These observations may provide a more precise view, but relatively few studies have used these markers. Moreover, more *in vitro* and animal-based studies are still required to understand the exact dynamics.

Most likely, methodological aspects have had an effect on the magnitude of the effect seen in some studies, or on their likelihood to be published, but they are not solely responsible for study outcome. This is especially likely in the presence of evidence from *in vitro* and mouse studies, in which T lymphocytes are not evaluated on a statistical, but on a mechanical level. Nonetheless, the importance of differences in methodology and/or reporting was highlighted by Altman *et al* (1995) and McShane *et al* (2005). They proposed guidelines for the reporting of prognostic marker studies, to encourage transparent reporting and to assist the reader in judging study quality (McShane *et al*, 2005). We used these guidelines in an adapted form to assess study quality (de Graeff *et al*, 2009). Using these criteria, we observed that 19 out of 52 studies (36.5%) failed to adequately report follow-up time and 22 out of 52 (42.3%) not report clear in- and exclusion criteria. Importantly, only one of the included studies reached the maximum score of 8 points (Nosho *et al*, 2010), mainly because all but two studies (Sinicrope *et al*, 2009; Nosho *et al*, 2010) were retrospective. Hoppin *et al* (2002) described nicely how retrospective tissue-based studies can lead to bias, as the availability of tumour specimens in pathology archives may depend on a wide variety of factors such as patient age, tumour size, tumour grade and hospital, in which the patient was diagnosed. An alternative to prospective studies is the structured collection of specimens from all patients and all hospitals in a region, and a very thorough description of patient demographics to analyse potential bias afterwards.

Our systematic review has some limitations of its own, inherent to its design. We included a very heterogeneous group of studies, consisting of many different types of cancer, different patient selection criteria and different types of methodology. As mentioned above, we felt it was justified to pool them anyway, because we expected the biological functions of TILs to be independent of tumour type. Nonetheless, the effect size that TILs might have in terms of prolonging survival, is undoubtedly related to clinicopathological factors such as differentiation grade. These interactions can be corrected for in multivariate analyses. However, multivariate analysis is not suitable for pooled analysis, because the covariates used in multivariate analysis vary between studies, and because their outcomes cannot be estimated based on other data in the paper.

Another limitation is that many studies had to be excluded from meta-analysis because they did not report HRs and CIs, but only Kaplan–Meier curves and log-rank tests. Only 9 out of 49 (18.4%) reported HRs and CIs for some or all stainings. We managed to reduce the missing data by contacting authors, and by estimating outcome with the help of a spreadsheet (Tierney *et al*, 2007). The latter may have introduced some imprecision, but we felt this was a risk worth taking in view of the alternative, that is, excluding the studies. This highlights the importance of a uniform reporting of study outcomes and follow-up time.

A final limitation is our use of strict inclusion criteria, which resulted in exclusion of smaller studies. This was intended to eliminate studies with little precision, but thereby also reduced the number of studies included in this meta-analysis because small studies are published relatively frequently. Hence, stratified analysis was not possible for all TIL subsets. Moreover, stratified analyses were performed in relatively limited numbers of studies. The results from these analyses should therefore not be interpreted strictly based on their numerical outcome, but rather as general suggestions for designing future studies.

In conclusion, we found evidence that TILs moderately influence prognosis, but this influence is more pronounced in studies incorporating lymphocyte ratios. However, the exact magnitude of TILs on prognosis remains somewhat mysterious due to methodological factors. Improving study quality is an essential step toward uncovering the real clinical relevance of TILs. Moreover, just quantifying TILs may not take the dynamics of the tumour microenvironment into account. Any future studies should have a very strict design, with large sample sizes to increase statistical power, a uniform way of analyzing survival outcomes, and a long and specified follow-up period.

## Figures and Tables

**Table 1:** Characteristics of the included studies

	**Number of studies**	**Number of patients**
*Types of cancer*		
Ovarian	14 (26.9%)	2574 (20.7%)
Colorectal	10 (19.2%)	3984 (32.0%)
Lung	7 (13.5%)	2328 (18.7%)
Hepatocellular	5 (9.6%)	909 (7.3%)
Renal cell	3 (5.8%)	416 (3.3%)
Other	13 (25.0%)	2234 (18.0%)
		
*Types of lymphocytes* a		
CD3+	22 (20.0%)	4678 (37.6%)
CD4+	15 (13.6%)	2707 (21.8%)
CD8+	32 (29.1%)	9126 (73.3%)
FoxP3+	23 (20.9%)	4786 (38.5%)
Ratios	18 (16.3%)	3017 (24.2%)
		
*Type of tissue analysed*		
Tissue microarray	20 (38.5%)	6487 (52.1%)
Whole-tissue slides	32 (61.5%)	5958 (47.9%)
		
*Areas of scoring*		
Hotspots	15 (28.8%)	2651 (21.3%)
Representative areas	37 (71.2%)	9794 (78.7%)

a Total percentage is >100%, as most studies included >1 lymphocyte subset, percentage of total numbers of studies and total number of patients included.

**Table 2:** Summarised hazard ratios

	**Number of studies**	**Number of patients**	**Pooled HR (95% CI)**	**I^2^ value (%)**	**P-value**	**P-value differences** a
*CD3*+						
Smallest studies^b^	7	1039	0.48 (0.33–0.69)	72	**<0.0001**	**<0.00001**
Largest studies^c^	4	1985	0.76 (0.55–1.07)	86	0.11	
Median follow-up <4 years	5	918	0.53 (0.36–0.78)	65	**0.001**	**<0.000001**
Median follow-up >4 years	4	1808	0.83 (0.66–1.04)	59	0.11	
Quality score ⩽5	4	741	0.58 (0.30–1.13)	89	0.11	0.11
Quality score >5	7	2301	0.58 (0.41–0.82)	86	**0.002**	
Tissue microarray	5	2223	0.70 (0.51–0.97)	85	**0.03**	**<0.00001**
Whole-tissue slides	6	819	0.48 (0.31–0.75)	77	**0.001**	
Hotspots scored	4	671	0.62 (0.37–1.04)	79	0.07	0.73
Representative areas scored	7	2371	0.55 (0.37–0.83)	89	**0.004**	
Ovarian cancer	5	1053	0.53 (0.28–1.01)	92	**0.05**	0.25
Colorectal cancer	3	1319	0.64 (0.37–1.10)	84	0.11	
						
*CD8*+						
Smallest studies^d^	12	2319	0.66 (0.53–0.81)	59	**0.0001**	**0.003**
Largest studies^e^	4	3740	0.82 (0.75–0.91)	0	**<0.0001**	
Median follow-up <4 years	6	1322	0.53 (0.45–0.62)	0	**<0.00001**	**0.0003**
Median follow-up >4 years	9	2539	0.76 (0.64–0.90)	48	**0.002**	
Quality score ⩽5	7	2348	0.74 (0.61–0.91)	48	**0.004**	0.46
Quality score >5	9	3693	0.69 (0.56–0.86)	70	**0.0006**	
Tissue microarray	9	3926	0.71 (0.60–0.85)	61	**0.0001**	0.53
Whole-tissue slides	7	2133	0.72 (0.55–0.95)	66	**0.02**	
Hotspots scored	5	893	0.75 (0.59–0.97)	37	**0.03**	0.98
Representative areas scored	11	5166	0.70 (0.58–0.83)	69	**<0.0001**	
Ovarian cancer	4	983	0.69 (0.54–0.88)	49	**0.003**	0.45
Colorectal cancer	4	2582	0.62 (0.44–0.88)	81	**0.007**	

Abbreviations: CIs=confidence intervals; HRs=hazard ratios.

All analyses were carried out for overall survival only. Median follow-up was not available for all studies.

a *P*-values for differences between pairs, bold indicating whether there are statistically significant differences between pooled results from, for instance, small and large studies.

b *n*=100–220.

c *n*=302–786.

d *n*=109–359.

e *n*=500–1290.
