# Supplementary material for: The prognostic influence of tumour-infiltrating lymphocytes in cancer: a systematic review with meta-analysis
Source: Br J Cancer. 2011 May 31;105(1):93–103. doi: 10.1038/bjc.2011.189 (PMC3137407; doi:10.1038/bjc.2011.189)
Supplement: Supplementary Information [file bjc2011189x1.doc]

**Supplementary tables Meta-analysis TIL in cancer**

Gooden et al.

| **Supplementary table 1** Criteria for quality assessment by De Graeff [de Graeff et al., 2009]. | | |
| --- | --- | --- |
| **Criterium** | **Points** | |
| 1. Is the population under study defined with in- and exclusion criteria? |  | **1** |
| 2. Were patient data prospectively collected? |  | **1** |
| 3. Are the main prognostic patient and tumour characteristics presented?^1^ |  | **1** |
| 4. Is the method used for determination of protein expression specified? |  | **2** |
| Criteria for immuhistochemistry / FISH:   - Is the immunohistochemical staining protocol specified?^2^ - Were stainings evaluated by > 1 observer? | 1  1 |  |
| Criteria for mutational analysis:   - Is the PCR protocol specified?^3^ - Is the SSCP and/or sequencing protocol specified? | 1  1 |  |
| Criteria for Southern Blot:   - Are the restriction enzymes used specified? - Is the hybridization methods specified?^4^ | 1  1 |  |
| Criteria for EGF binding assay:   - Are positive and negative controls specified? - Is the assay protocol specified?^5^ | 1  1 |  |
| Criteria for RT-PCR:   - Is the RNA isolation method and cDNA synthesis specified? - Is the PCR protocol specified?^3^ | 1  1 |  |
| Criteria for enzyme immunoassay   - Is the antibody used specified? - Are control samples and a cut-off value specified? | 1  1 |  |
| 5. Is the study endpoint defined? |  | **1** |
| 6. Is the time of follow up specified? |  | **1** |
| 7. Is loss during analysis or follow up described? |  | **1** |
|  | **Max. 8 points** | |
| ^1^At least four of the following characteristics: age at diagnosis, FIGO stage, tumor type, differentiation grade and residual tumor after primary surgery. ^2^At least four of the following criteria: antigen retrieval, primary antibody, dilution, detection method, cut-off value for positive expression: ^3^At least the primers used and the annealing temperature or number of cycles ^4^At least internal controls and probes used ^5^At least four of the following criteria: label, incubation time, filter size, separation method (BSA / Tris-sucrose), cut-off value for positive expression | | |

| **Supplementary Table 2** CD3+ tumor infiltrating lymphocytes | | | | | | | | |
| --- | --- | --- | --- | --- | --- | --- | --- | --- |
| **Author** | **Tumor site** | **N** | **Selection** | **Follow up** | **Cutoff** | **Outcome** | **Univariate analysis** | **Multivariate analysis** |
| [Nedergaard et al., 2007] | Cervix | 102 | Stage IB & IIA | 64 | 75th percentile | RFS better | 0.49 (0.28-0.86) p≤0.05 | n.s. |
| [Nosho et al., 2010] | CRC | 768 |  | 139 | lowest vs. highest quartile | OS =  DSS = | 0.94 (0.70-1.26) n.s.  0.73 (0.49-1.08) n.s. | 1.37 (0.96-1.96) n.s.  1.30 (0.81-2.07) n.s. |
| [Galon et al., 2006]* | CRC | 415 |  | 45 | 370/mm^2^ | OS better  DFS better | 0.44 (0.32-0.59) p≤0.001  0.47 (0.34-0.65) p≤0.001 | 0.27 (0.14-0.51) p≤0.001 |
| [Simpson et al., 2010] | CRC | 355 |  | 42 | mean | DSS better | p≤0.05 | 0.63 (0.43-0.93) p≤0.05 |
| [Deschoolmeester et al., 2010] | CRC | 215 |  | 62 | 20 per 1.19mm^2^ | OS better  DFS = | p≤0.05 | 0.54 (0.18-1.59) n.s.  1.61 (0.44-5.95) n.s. |
| [Sinicrope et al., 2009]* | CRC | 136 | Stage II & III |  | 25th percentile | DFS better  OS = | 0.54 (0.32-0.91) p≤0.05  0.63 (0.41-1.15) n.s. | 0.56 (0.32-0.96) p≤0.05 |
| [Baeten et al., 2006] | CRC | 117 |  |  | unknown | OS = | n.s. |  |
| [Zingg et al., 2010] | Esophageal | 130 |  |  | median | OS better | 0.53 (0.33-0.84) p≤0.05 | 1.29 (0.56-2.97) n.s. |
| [Lee et al., 2008] | Gastric | 220 |  | 64 | 75th percentile | OS better | 0.45 (0.26-0.76) p≤0.05 | 0.55 (0.32-0.95) p≤0.05 |
| [Gao et al., 2007] | HCC | 302 |  | 58 | median | OS =  DFS = | 0.84 (0.63-1.12) n.s.  0.84 (0.62-1.14) n.s. |  |
| [Cai et al., 2006] | HCC | 173 |  |  | unknown | DFS better |  | p≤0.05 |
| [Toomey et al., 2003] | NSCLC | 117 |  |  | present | OS =  PFS better | n.s.  p≤0.001 | 0.17 (0.08-0.36) p=? |
| [Clarke et al., 2009] | Ovarian | 500 | Stage I, II, III | 63 | present | OS = | 0.94 (0.81-1.11) n.s. |  |
| [Zhang et al., 2003]* | Ovarian | 186 | Stage III, IV |  | present | OS better  PFS better | 0.24 (0.16-0.36) p≤0.001  0.36 (0.23-0.56) p≤0.001 | p≤0.05 |
| [Al Attar et al., 2010] | Ovarian | 157 |  | 36 | mean | OS better  DFS = | p≤0.05  n.s. |  |
| [Adams et al., 2009]* | Ovarian | 134 | Stage III, IV | 42 | present | OS = | 0.67 (0.33-1.35) n.s. | 0.53 (?-?) n.s. |
| [Shah et al., 2008] | Ovarian | 119 |  |  | low - medium - high | OS = | n.s. |  |
| [Sato et al., 2005] | Ovarian | 117 |  | 30 | 33rd percentile | OS = | 0.92 (0.54-1.55) n.s. | 0.71 (0.36-1.29) n.s. |
| [Tomsova et al., 2007]* | Ovarian | 116 |  | 39 | median | OS better | 0.30 (0.18-0.49) p≤0.001 | 0.27 (0.15-0.50) p≤0.001 |
| [Stumpf et al., 2009] | Ovarian | 100 | Stage III, serous | 23 | 0 vs. <5 vs. >5 | OS =  DFS better | n.s.  p≤0.05 |  |
| [Sorbye et al., 2011] | STS | 249 |  | 38 | unknown | DSS = | 1.47 (0.97-2.23) n.s.^‡^ |  |
| Follow up: median follow up in months. All hazard ratios are defined as high TIL versus low TIL. Abbreviations: CRC: colorectal cancer; HCC: hepatocellular cancer; NSCLC: non small cell lung cancer; STS: soft tissue sarcoma; OS: overall survival; DFS: disease free survival; PFS: progression free survival; RFS: relapse free survival, n.s.: not significant. * Hazard ratio and/or confidence interval estimated based on Tierney et al. [Tierney et al., 2007] | | | | | | | | |

| **Supplementary Table 3** CD4+ tumor infiltrating lymphocytes | | | | | | | | |
| --- | --- | --- | --- | --- | --- | --- | --- | --- |
| **Author** | **Tumor site** | **N** | **Selection** | **Follow up** | **Cutoff** | **Outcome** | **Univariate analysis** | **Multivariate analysis** |
| [Jordanova et al., 2008] | Cervix | 115 | Stage Ib, II | 60 | median | OS = | n.s. |  |
| [Nedergaard et al., 2007] | Cervix | 102 | Stage Ib, IIa |  | 75th percentile | RFS better | 0.55 (0.31-1.00) p≤0.05 |  |
| [Zingg et al., 2010] | Esophageal | 130 |  |  | median | OS = | 0.74 (0.47-1.16) n.s. | 0.62 (0.28-1.37) n.s. |
| [Cho et al., 2003] | Esophageal | 122 |  |  | median | OS better | p≤0.05 |  |
| [Shen et al., 2010] | Gastric | 133 |  | 43 | median | OS = | 0.80 (0.42-1.52) n.s. |  |
| [Gao et al., 2007] | HCC | 302 |  | 58 | median | OS =  DFS = | 0.82 (0.62-1.10) n.s.  0.89 (0.65-1.20) n.s. |  |
| [Al Shibli et al., 2008] | NSCLC | 335 | Stage I-IIIa | 96 | ≥5% of all cells | DSS = | 0.86 (0.59-1.27) n.s.* |  |
| [Wakabayashi et al., 2003] | NSCLC | 178 | Stage I-IIIa |  | median | OS = | n.s. |  |
| [Clarke et al., 2009] | Ovarian | 500 | Stage I, II, III | 63 | present | OS = | 0.92 (0.62-1.32) n.s.^‡^ | 0.72 (0.48-1.05) n.s.^‡^ |
| [Sato et al., 2005] | Ovarian | 117 |  | 30 | 33rd percentile | OS = | 0.84 (0.50-1.40) n.s. |  |
| [Stumpf et al., 2009] | Ovarian | 100 | Stage III, serous | 23 | 0 vs. <5 vs. >5 | OS =  DFS = | n.s.  n.s. |  |
| [Li et al., 2009] | RCC | 125 |  | 57 | median | OS =  DFS = | 0.68 (0.31-1.48) n.s.  0.68 (0.32-1.50) n.s. |  |
| [Sorbye et al., 2011] | STS | 249 |  | 38 | unknown | DSS = | 0.66 (0.38-1.15) n.s.^‡^ |  |
| Follow up: median follow up in months. All hazard ratios are defined as high TIL versus low TIL. Abbreviations: HCC: hepatocellular cancer; NSCLC: non small cell lung cancer; RCC: renal cell cancer; OS: overall survival; DSS: disease specific survival; DFS: disease free survival; RFS: relapse free survival, n.s.: not significant. * Hazard ratio and/or confidence interval estimated based on Tierney et al. [Tierney et al., 2007], ^‡^ Hazard ratio and/or confidence interval acquired from the authors | | | | | | | | |

| **Supplementary Table 4** CD8+ tumor infiltrating lymphocytes | | | | | | | | |
| --- | --- | --- | --- | --- | --- | --- | --- | --- |
| **Author** | **Tumor site** | **N** | **Selection** | **Follow up** | **Cutoff** | **Outcome** | **Univariate analysis** | **Multivariate analysis** |
| [Jordanova et al., 2008] | Cervix | 115 | Stage Ib, II | 60 | median | OS = | 0.94 (0.40-2.19) n.s.^‡^ |  |
| [Nedergaard et al., 2007] | Cervix | 102 | Stage Ib, IIa |  | 75th percentile | DFS better | 0.41 (0.21-0.80) p≤0.05 | n.s. |
| [Zlobec et al., 2007] | CRC | 1164 |  |  | >4/TMA core | OS better | 0.86 (0.51-0.74) p≤0.001 |  |
| [Chiba et al., 2004] | CRC | 371 |  | 89 | median | DSS better | 0.41 (0.29-0.59) p≤0.001^‡^ | 0.71 (0.48-1.00) p≤0.05 |
| [Galon et al., 2006] | CRC | 359 |  | 45 | 80/mm^2^ | OS better  PFS better | 0.44 (0.32-0.60) p≤0.001*  0.46 (0.33-0.65) p≤0.001* |  |
| [Nosho et al., 2010] | CRC | 768 |  | 139 | lowest vs. highest quartile | OS better  DSS better | 0.74 (0.54-0.99) p≤0.05  0.61 (0.42-0.88) p≤0.05 | 0.85 (0.60-1.20) n.s.  0.81 (0.52-1.27) n.s. |
| [Kasajima et al., 2010] | CRC | 291 |  | 38 | none/mild vs. moderate/severe | OS better | 0.48 (0.29-0.78) p≤0.05* | 0.63 (0.34-1.19) n.s. |
| [Deschoolmeester et al., 2010] | CRC | 215 |  | 62 | 20 per 1.19mm^2^ | OS better  DFS = | p≤0.05 | 2.06 (0.67-6.39) n.s.  0.68 (0.16-2.88) n.s. |
| [Prall et al., 2004] | CRC | 152 | Stage III | 43 | 66th percentile | DSS better  PFS better | 0.54 (0.30-0.95) p≤0.05^‡^  0.43 (0.22-0.85) p≤0.05^‡^ | 0.43 (0.22-0.85) p≤0.05  0.56 (0.37-0.99) p≤0.05 |
| [Baeten et al., 2006] | CRC | 117 |  |  | unknown | OS better | p≤0.05 |  |
| [De Jong et al., 2009] | Endometrial | 368 |  |  | 20th percentile | DSS better  DFS better | 0.31 (0.18-0.55) p≤0.05^‡^  0.55 (0.33-0.92) p≤0.05^‡^ | p≤0.05  n.s. |
| [Zingg et al., 2010] | Esophageal | 130 |  |  | median | OS better | 0.44 (0.27-0.69) p≤0.001 | 0.62 (0.28-1.37) n.s. |
| [Cho et al., 2003] | Esophageal | 122 |  |  | median | OS better | p≤0.05 |  |
| [Lee et al., 2008] | Gastric | 220 |  | 64 | 75th percentile | OS better | 0.58 (0.36-0.95) p≤0.05 | 0.57 (0.37-0.95) p≤0.05 |
| [Shen et al., 2010] | Gastric | 133 |  | 43 | median | OS = | 0.80 (0.42-1.52) n.s. |  |
| [Gao et al., 2007] | HCC | 302 |  | 58 | median | OS =  DFS = | 0.84 (0.63-1.12) n.s.  0.79 (0.58-1.07) n.s. |  |
| [Cai et al., 2006] | HCC | 17l3 |  |  | unknown | DFS better |  | p≤0.05 |
| [Ruffini et al., 2009] | NSCLC | 1290 | Stage I-IIIa |  | present | OS better | 0.78 (0.64-0.98) n.s.^‡^ | 0.78 (0.64-0.98) p≤0.05 |
| [Al Shibli et al., 2008] | NSCLC | 335 | Stage I-IIIa | 96 | >5% | DSS better | 0.64 (0.44-0.94) p≤0.05* | n.s. |
| [Kawai et al., 2008] | NSCLC | 199 | Stage IV |  | median | OS better | p≤0.001 | p≤0.001 |
| [Wakabayashi et al., 2003] | NSCLC | 178 | Stage I-IIIa |  | median | OS worse | p≤0.05 | n.s. |
| [Hiraoka et al., 2006a] | NSCLC | 109 |  |  | mean | OS = | 1.23 (0.68-2.22) n.s.^‡^ | 0.80 (0.41-1.57) n.s.^‡^ |
| [Clarke et al., 2009] | Ovarian | 500 | Stage I, II, III | 63 | present | OS better | 0.84 (0.72-0.98) p≤0.05 | 0.75 (0.61-0.93) p≤0.05 |
| [Leffers et al., 2009] | Ovarian | 270 |  |  | 33rd percentile | DSS better | 0.68 (0.46-0.99) p≤0.05^‡^ | 0.35 (0.21-0.60) p≤0.001 |
| [Barnett et al., 2010] | Ovarian | 232 | Advanced | 47 | ≥2.1/HPF | OS better | 0.63 (0.44-0.90) p≤0.05^‡^ |  |
| [Callahan et al., 2008] | Ovarian | 184 | Stage IIIb-IV |  | 75th percentile | OS better | p≤0.05 | 0.58 (0.37-0.89) p≤0.05 |
| [Adams et al., 2009] | Ovarian | 134 | Stage III-IV | 42 | >10/HPF | OS better | 0.59 (0.36-1.01) n.s.* | 0.50 (?-?) p≤0.05 |
| [Sato et al., 2005] | Ovarian | 117 |  | 30 | 33rd percentile | OS better | 0.51 (0.30-0.85) p≤0.05 | 0.33 (0.18-0.60) p≤0.001 |
| [Stumpf et al., 2009] | Ovarian | 100 | Stage III, serous |  | 0 vs. <5 vs. >5 | OS better  DFS = | p≤0.05  n.s. | 0.41 (0.25-0.68) p≤0.001 |

| [Sorbye et al., 2011] | STS | 249 |  | 38 | unknown | DSS = | 0.95 (0.59-1.52) n.s.^‡^ |  |
| --- | --- | --- | --- | --- | --- | --- | --- | --- |
| [Jensen et al., 2009] | RCC | 121 |  | 124 | median | RFS =  DSS =  OS = | 1.29 (0.77-1.94) n.s.^‡^  1.43 (0.84-2.45) n.s.^‡^  1.22 (0.77-1.94) n.s.^‡^ | 1.23 (0.70-2.20) n.s.^‡^  1.34 (0.73-2.70) n.s.^‡^  1.2 (0.70-2.04) n.s.^‡^ |
| Follow up: median follow up in months. All hazard ratios are defined as high TIL versus low TIL. Abbreviations: CRC: colorectal cancer; HCC: hepatocellular cancer; NSCLC: non small cell lung cancer; RCC: renal cell cancer; OS: overall survival; DSS: disease specific survival; DFS: disease free survival; PFS: progression free survival; RFS: relapse free survival, n.s.: not significant. * Hazard ratio and/or confidence interval estimated based on Tierney et al. [Tierney et al., 2007], ^‡^ Hazard ratio and/or confidence interval acquired from the authors | | | | | | | | |

| **Supplementary Table 5** FoxP3+ tumor infiltrating lymphocytes | | | | | | | | |
| --- | --- | --- | --- | --- | --- | --- | --- | --- |
| **Author** | **Tumor site** | **N** | **Selection** | **Follow up** | **Cutoff** | **Outcome** | **Univariate analysis** | **Multivariate analysis** |
| [de Kruijf et al., 2010] | Breast | 356 | No systemic therapy | 228 | presence | OS =  RFS = | n.s.  n.s. |  |
| [Bates et al., 2006] | Breast | 283 |  | 87 | median | OS worse  RFS worse | 1.98 (1.19-3.30) p≤0.05^‡^  1.85 (1.21-2.84) p≤0.05^‡^ | 1.62 (0.96-2.74) n.s.  1.58 (1.01-2.47) p≤0.05 |
| [Gobert et al., 2009] | Breast | 191 | M0 | 84 | 75th percentile | OS = | n.s. |  |
| [de Kruijf et al., 2010] | Breast | 129 | Systemic therapy | 228 | presence | OS =  RFS better | n.s.  0.60 (0.35-1.02) n.s. | 0.49 (0.28-0.86) p≤0.05 |
| [Jordanova et al., 2008] | Cervix | 115 | Stage Ib-II |  | median | DSS worse | 2.76 (1.01-7.55) p≤0.05 | 2.25 (0.67-7.50) n.s. |
| [Nosho et al., 2010] | CRC | 768 |  | 139 | lowest vs. highest quartile | OS better  DSS better | 0.48 (0.35-0.66) p≤0.001  0.48 (0.32-0.70) p≤0.001 | 0.80 (0.57-1.14) n.s.  0.89 (0.59-1.34) n.s. |
| [Sinicrope et al., 2009] | CRC | 136 | Stage II-III |  | 25th percentile | OS =  DFS = | 1.37 (0.81-2.33) n.s.  1.24 (0.72-2.13) n.s. |  |
| [De Jong et al., 2009] | Endometrial | 368 |  |  | >8/TMA core | DSS =  DFS = | 1.04 (0.52-2.08) n.s.^‡^  1.19 (0.66-2.13) n.s.^‡^ |  |
| [Zingg et al., 2010] | Esophageal | 130 |  |  | median | OS = | 0.65 (0.40-1.05) n.s. |  |
| [Shen et al., 2010] | Gastric | 133 |  | 43 | median | OS = | 0.47 (0.24-0.93) p≤0.05 | 0.86 (0.39-1.93) n.s. |
| [Heimberger et al., 2008] | Glioma | 135 |  |  | presence | OS better | p≤0.001 |  |
| [Gao et al., 2007] | HCC | 302 |  | 58 | median | OS worse  DSS worse | 1.50 (1.10-2.00) p≤0.05  1.40 (1.00-2.00) p≤0.05 | 1.69 (1.20-2.33) p≤0.001  1.14 (0.93-1.92) n.s. |
| [Sasaki et al., 2008] | HCC | 164 |  | 55 | median | DSS =  DFS worse | 1.22 (0.75-1.97) n.s.^‡^  1.23 (1.03-1.47) p≤0.05^‡^ | 1.12 (1.01-1.45) p≤0.05 |
| [Shimizu et al., 2010] | NSCLC | 100 |  |  | median | RFS worse | p≤0.05 | 2.02 (0.86-4.74) n.s. |
| [Leffers et al., 2009] | Ovarian | 270 |  |  | presence | DSS = | 0.89 (0.62-1.29) n.s.^‡^ |  |
| [Milne et al., 2009] | Ovarian | 500 | High grade, serous |  | presence | DSS = | 1.13 (0.79-1.64) n.s.^‡^ |  |
| [Barnett et al., 2010] | Ovarian | 232 | Advanced | 47 | ≥20/HPF | OS = | 1.11 (0.68-1.83) n.s.^‡^ |  |
| [Adams et al., 2009] | Ovarian | 134 | Stage III, IV | 42 | >10/HPF | OS worse | 1.50 (0.77-2.76) n.s.* | 2.10 (?-?) p≤0.05 |
| [Shah et al., 2008] | Ovarian | 119 |  |  | presence | OS = | n.s. |  |
| [Sato et al., 2005] | Ovarian | 117 |  | 30 | 33rd percentile | OS = | 1.28 (0.74-2.24) n.s. | 1.31 (0.72-2.41) n.s. |
| [Perrone et al., 2008] | Ovarian | 110 | Stage II-III |  | median | OS worse  RFS worse | 2.41 (1.37-4.25) p≤0.05*  2.15 (1.22-3.78) p≤0.05* | 2.34 (1.27-4.28) p≤0.05  2.00 (1.10-3.65) p≤0.05 |
| [Siddiqui et al., 2007] | RCC | 170 |  | 44 | presence | DSS = | 1.28 (0.63-2.59) n.s. | 1.20 (0.59-2.44) n.s. |
| [Li et al., 2009] | RCC | 125 |  | 57 | median | OS =  DFS= | 1.38 (0.64-2.97) n.s.  1.37 (0.64-2.94) n.s. |  |
| Follow up: median follow up in months. All hazard ratios are defined as high TIL versus low TIL. Abbreviations: CRC: colorectal cancer; HCC: hepatocellular cancer; RCC: renal cell cancer; TMA: tissue microarray; HPF: high power field; OS: overall survival; DSS: disease specific survival; DFS: disease free survival; PFS: progression free survival; RFS: relapse free survival, n.s.: not significant. * Hazard ratio and/or confidence interval estimated based on Tierney et al. [Tierney et al., 2007], ^‡^ Hazard ratio and/or confidence interval acquired from the authors | | | | | | | | |

| **Supplementary Table 6** Ratios | | | | | | | | | |
| --- | --- | --- | --- | --- | --- | --- | --- | --- | --- |
| **Author** | **Ratio** | **Tumor site** | **Comparison** | **N** | **Selection** | **Follow up** | **Outcome** | **Univariate analysis** | **Multivariate analysis** |
| [Han et al., 2008] | Ovarian | CD3/CD8 | CD3 or CD8 vs. none | 150 |  | 22 | DSS better | 0.49 (0.33-0.74) p≤0.05 | 0.57 (0.34-0.94) p≤0.05 |
| [Lee et al., 2008] | Gastric | CD3/CD8 | hihi vs. lolo | 220 |  | 64 | OS better | 0.37 (0.19-0.72) p≤0.05 | 0.40 (0.21-0.80) p≤0.05 |
| [Kobayashi et al., 2007] | HCC | CD3/CD8 | high vs. low | 147 |  | 52 | OS = | 0.95 (0.62-1.46) n.s. | 0.90 (0.55-1.47) n.s. |
|  | | | | | | | | | |
| [Jordanova et al., 2008] | Cervix | CD8/CD4 | high vs. low | 115 | Stage Ib-II | 60 | OS = | n.s. |  |
| [Zingg et al., 2010] | Esophageal | CD8/CD4 | high vs. low | 130 |  |  | OS = | 0.71 (0.43-1.19) n.s. |  |
| [Sato et al., 2005] | Ovarian | CD8/CD4 | high vs. low | 117 |  | 30 | OS better | 0.42 (0.25-0.72) p≤0.05 | 0.30 (0.16-0.55) p≤0.001 |
|  | | | | | | | | | |
| [Jordanova et al., 2008] | Cervix | CD8/FoxP3 | high vs. low | 115 | Stage Ib-II | 60 | OS better | 0.36 (0.14-0.92) p≤0.05 | 0.37 (0.14-0.99) p≤0.05 |
| [De Jong et al., 2009] | Endometrial | CD8/FoxP3 | high vs. low | 368 |  |  | DSS better  DFS better | 0.20 (0.10-0.41) p≤0.001^‡^  0.49 (0.33-0.83) p≤0.05^‡^ | n.s.  n.s. |
| [Zingg et al., 2010] | Esophageal | CD8/FoxP3 | high vs. low | 130 |  |  | OS = | 0.84 (0.51-1.38) n.s. |  |
| [Shen et al., 2010] | Gastric | CD8/FoxP3 | high vs. low | 133 |  | 43 | OS better | 0.30 (0.13-0.67) p≤0.05 | 0.35 (0.13-0.94) p≤0.05 |
| [Cai et al., 2009] | HCC | CD8/FoxP3 | high vs. low | 173 |  | 37 | OS better  RFS better | 0.42 (0.27-0.66)p≤0.001^‡^  0.41 (0.27-0.63)p≤0.001^‡^ | 0.50 (0.31-0.79) p≤0.05  0.47 (0.30-0.73) p≤0.05 |
| [Leffers et al., 2009] | Ovarian | CD8/FoxP3 | high vs. low | 270 |  |  | DSS better | 0.62 (0.42-0.91) p≤0.05^‡^ | 0.53 (0.32-0.85) p≤0.05 |
| [Barnett et al., 2010] | Ovarian | CD8/FoxP3 | high vs. low | 232 | Advanced | 47 | OS = | 0.66 (0.39-1.12) n.s.^‡^ |  |
| [Shah et al., 2008] | Ovarian | CD8/FoxP3 | high vs. low | 119 |  |  | OS = | n.s. |  |
| [Sato et al., 2005] | Ovarian | CD8/FoxP3 | high vs. low | 117 |  | 30 | OS better | 0.33 (0.18-0.59) p≤0.001 | 0.31 (0.17-0.58) p≤0.001 |
|  | | | | | | | | | |
| [Hiraoka et al., 2006b] | Pancreas | FoxP3/CD4 | high vs. low | 198 |  | 20 | DSS worse | 2.56 (1.77-3.70) p≤0.001 | 2.45 (1.62-3.72) p≤0.001 |
| [Kobayashi et al., 2007] | HCC | FoxP3/CD4 | high vs. low | 147 |  | 52 | OS worse  DFS worse | 1.79 (1.16-2.76) p≤0.05  1.70 (1.11-2.62) p≤0.05 | 1.64 (1.02-2.63) p≤0.05  1.71 (1.07-2.71) p≤0.05 |
|  | | | | | | | | | |
| [Sinicrope et al., 2009] | CRC | CD3/FoxP3 | high vs. low | 136 | Stage II-III |  | OS =  DFS better | 0.57 (0.30-1.09) n.s.  0.46 (0.24-0.92) p≤0.05 | 0.45 (0.23-0.91) p≤0.05 |
| Follow up: median follow up in months. All hazard ratios are defined as high TIL versus low TIL. Abbreviations: CRC: colorectal cancer; HCC: hepatocellular cancer; RCC: renal cell cancer; OS: overall survival; DSS: disease specific survival; DFS: disease free survival; PFS: progression free survival; RFS: relapse free survival, n.s.: not significant. ^‡^ Hazard ratio and/or confidence interval acquired from the authors | | | | | | | | | |

Reference List

Adams SF, Levine DA, Cadungog MG, Hammond R, Facciabene A, Olvera N, Rubin SC, Boyd J, Gimotty PA, Coukos G (2009) Intraepithelial T cells and tumor proliferation: impact on the benefit from surgical cytoreduction in advanced serous ovarian cancer. *Cancer* **115**: 2891-2902

Al Attar A, Shehata M, Durrant L, Moseley P, Deen S, Chan S (2010) T cell density and location can influence the prognosis of ovarian cancer. *Pathol Oncol Res* **16**: 361-370

Al Shibli KI, Donnem T, Al Saad S, Persson M, Bremnes RM, Busund LT (2008) Prognostic effect of epithelial and stromal lymphocyte infiltration in non-small cell lung cancer. *Clin Cancer Res* **14**: 5220-5227

Baeten CI, Castermans K, Hillen HF, Griffioen AW (2006) Proliferating endothelial cells and leukocyte infiltration as prognostic markers in colorectal cancer. *Clin Gastroenterol Hepatol* **4**: 1351-1357

Barnett JC, Bean SM, Whitaker RS, Kondoh E, Baba T, Fujii S, Marks JR, Dressman HK, Murphy SK, Berchuck A (2010) Ovarian cancer tumor infiltrating T-regulatory (T(reg)) cells are associated with a metastatic phenotype. *Gynecol Oncol* **116**: 556-562

Bates GJ, Fox SB, Han C, Leek RD, Garcia JF, Harris AL, Banham AH (2006) Quantification of regulatory T cells enables the identification of high-risk breast cancer patients and those at risk of late relapse. *J Clin Oncol* **24**: 5373-5380

Cai MY, Xu YF, Qiu SJ, Ju MJ, Gao Q, Li YW, Zhang BH, Zhou J, Fan J (2009) Human leukocyte antigen-G protein expression is an unfavorable prognostic predictor of hepatocellular carcinoma following curative resection. *Clin Cancer Res* **15**: 4686-4693

Cai XY, Gao Q, Qiu SJ, Ye SL, Wu ZQ, Fan J, Tang ZY (2006) Dendritic cell infiltration and prognosis of human hepatocellular carcinoma. *J Cancer Res Clin Oncol* **132**: 293-301

Callahan MJ, Nagymanyoki Z, Bonome T, Johnson ME, Litkouhi B, Sullivan EH, Hirsch MS, Matulonis UA, Liu J, Birrer MJ, Berkowitz RS, Mok SC (2008) Increased HLA-DMB expression in the tumor epithelium is associated with increased CTL infiltration and improved prognosis in advanced-stage serous ovarian cancer. *Clin Cancer Res* **14**: 7667-7673

Chiba T, Ohtani H, Mizoi T, Naito Y, Sato E, Nagura H, Ohuchi A, Ohuchi K, Shiiba K, Kurokawa Y, Satomi S (2004) Intraepithelial CD8+ T-cell-count becomes a prognostic factor after a longer follow-up period in human colorectal carcinoma: possible association with suppression of micrometastasis. *Br J Cancer* **91**: 1711-1717

Cho Y, Miyamoto M, Kato K, Fukunaga A, Shichinohe T, Kawarada Y, Hida Y, Oshikiri T, Kurokawa T, Suzuoki M, Nakakubo Y, Hiraoka K, Murakami S, Shinohara T, Itoh T, Okushiba S, Kondo S, Katoh H (2003) CD4+ and CD8+ T cells cooperate to improve prognosis of patients with esophageal squamous cell carcinoma. *Cancer Res* **63**: 1555-1559

Clarke B, Tinker AV, Lee CH, Subramanian S, van de Rijn M, Turbin D, Kalloger S, Han G, Ceballos K, Cadungog MG, Huntsman DG, Coukos G, Gilks CB (2009) Intraepithelial T cells and prognosis in ovarian carcinoma: novel associations with stage, tumor type, and BRCA1 loss. *Mod Pathol* **22**: 393-402

de Graeff P, Crijns AP, de Jong S, Boezen M, Post WJ, de Vries EG, van der Zee AG, de Bock GH (2009) Modest effect of p53, EGFR and HER-2/neu on prognosis in epithelial ovarian cancer: a meta-analysis. *Br J Cancer* **101**: 149-159

De Jong RA, Leffers N, Boezen HM, ten Hoor KA, van der Zee AG, Hollema H, Nijman HW (2009) Presence of tumor-infiltrating lymphocytes is an independent prognostic factor in type I and II endometrial cancer. *Gynecol Oncol* **114**: 105-110

de Kruijf EM, Sajet A, van Nes JG, Natanov R, Putter H, Smit VT, Liefers GJ, van den Elsen PJ, van de Velde CJ, Kuppen PJ (2010) HLA-E and HLA-G Expression in Classical HLA Class I-Negative Tumors Is of Prognostic Value for Clinical Outcome of Early Breast Cancer Patients. *J Immunol* **185**: 7452-7459

Deschoolmeester V, Baay M, Van Marck E, Weyler J, Vermeulen P, Lardon F, Vermorken JB (2010) Tumor infiltrating lymphocytes: an intriguing player in the survival of colorectal cancer patients. *BMC Immunol* **11**: 19

Galon J, Costes A, Sanchez-Cabo F, Kirilovsky A, Mlecnik B, Lagorce-Pages C, Tosolini M, Camus M, Berger A, Wind P, Zinzindohoue F, Bruneval P, Cugnenc PH, Trajanoski Z, Fridman WH, Pages F (2006) Type, density, and location of immune cells within human colorectal tumors predict clinical outcome. *Science* **313**: 1960-1964

Gao Q, Qiu SJ, Fan J, Zhou J, Wang XY, Xiao YS, Xu Y, Li YW, Tang ZY (2007) Intratumoral balance of regulatory and cytotoxic T cells is associated with prognosis of hepatocellular carcinoma after resection. *J Clin Oncol* **25**: 2586-2593

Gobert M, Treilleux I, Bendriss-Vermare N, Bachelot T, Goddard-Leon S, Arfl V, Biota C, Doffin AC, Durand I, Olive D, Perez S, Pasqual N, Faure C, Ray-Coquard I, Puisieux A, Caux C, Blay JY, Menetrier-Caux C (2009) Regulatory T cells recruited through CCL22/CCR4 are selectively activated in lymphoid infiltrates surrounding primary breast tumors and lead to an adverse clinical outcome. *Cancer Res* **69**: 2000-2009

Han LY, Fletcher MS, Urbauer DL, Mueller P, Landen CN, Kamat AA, Lin YG, Merritt WM, Spannuth WA, Deavers MT, De Geest K, Gershenson DM, Lutgendorf SK, Ferrone S, Sood AK (2008) HLA class I antigen processing machinery component expression and intratumoral T-Cell infiltrate as independent prognostic markers in ovarian carcinoma. *Clin Cancer Res* **14**: 3372-3379

Heimberger AB, Abou-Ghazal M, Reina-Ortiz C, Yang DS, Sun W, Qiao W, Hiraoka N, Fuller GN (2008) Incidence and prognostic impact of FoxP3+ regulatory T cells in human gliomas. *Clin Cancer Res* **14**: 5166-5172

Hiraoka K, Miyamoto M, Cho Y, Suzuoki M, Oshikiri T, Nakakubo Y, Itoh T, Ohbuchi T, Kondo S, Katoh H (2006a) Concurrent infiltration by CD8+ T cells and CD4+ T cells is a favourable prognostic factor in non-small-cell lung carcinoma. *Br J Cancer* **94**: 275-280

Hiraoka N, Onozato K, Kosuge T, Hirohashi S (2006b) Prevalence of FOXP3+ regulatory T cells increases during the progression of pancreatic ductal adenocarcinoma and its premalignant lesions. *Clin Cancer Res* **12**: 5423-5434

Jensen HK, Donskov F, Marcussen N, Nordsmark M, Lundbeck F, der Maase H (2009) Presence of intratumoral neutrophils is an independent prognostic factor in localized renal cell carcinoma. *J Clin Oncol* **27**: 4709-4717

Jordanova ES, Gorter A, Ayachi O, Prins F, Durrant LG, Kenter GG, van der Burg SH, Fleuren GJ (2008) Human leukocyte antigen class I, MHC class I chain-related molecule A, and CD8+/regulatory T-cell ratio: which variable determines survival of cervical cancer patients? *Clin Cancer Res* **14**: 2028-2035

Kasajima A, Sers C, Sasano H, Johrens K, Stenzinger A, Noske A, Buckendahl AC, Darb-Esfahani S, Muller BM, Budczies J, Lehman A, Dietel M, Denkert C, Weichert W (2010) Down-regulation of the antigen processing machinery is linked to a loss of inflammatory response in colorectal cancer. *Hum Pathol* **41**: 1758-1769

Kawai O, Ishii G, Kubota K, Murata Y, Naito Y, Mizuno T, Aokage K, Saijo N, Nishiwaki Y, Gemma A, Kudoh S, Ochiai A (2008) Predominant infiltration of macrophages and CD8(+) T Cells in cancer nests is a significant predictor of survival in stage IV nonsmall cell lung cancer. *Cancer* **113**: 1387-1395

Kobayashi N, Hiraoka N, Yamagami W, Ojima H, Kanai Y, Kosuge T, Nakajima A, Hirohashi S (2007) FOXP3+ regulatory T cells affect the development and progression of hepatocarcinogenesis. *Clin Cancer Res* **13**: 902-911

Lee HE, Chae SW, Lee YJ, Kim MA, Lee HS, Lee BL, Kim WH (2008) Prognostic implications of type and density of tumour-infiltrating lymphocytes in gastric cancer. *Br J Cancer* **99**: 1704-1711

Leffers N, Gooden MJ, De Jong RA, Hoogeboom BN, ten Hoor KA, Hollema H, Boezen HM, van der Zee AG, Daemen T, Nijman HW (2009) Prognostic significance of tumor-infiltrating T-lymphocytes in primary and metastatic lesions of advanced stage ovarian cancer. *Cancer Immunol Immunother* **58**: 449-459

Li JF, Chu YW, Wang GM, Zhu TY, Rong RM, Hou J, Xu M (2009) The prognostic value of peritumoral regulatory T cells and its correlation with intratumoral cyclooxygenase-2 expression in clear cell renal cell carcinoma. *BJU Int* **103**: 399-405

Milne K, Kobel M, Kalloger SE, Barnes RO, Gao D, Gilks CB, Watson PH, Nelson BH (2009) Systematic analysis of immune infiltrates in high-grade serous ovarian cancer reveals CD20, FoxP3 and TIA-1 as positive prognostic factors. *PLoS ONE* **4**:

Nedergaard BS, Ladekarl M, Thomsen HF, Nyengaard JR, Nielsen K (2007) Low density of CD3+, CD4+ and CD8+ cells is associated with increased risk of relapse in squamous cell cervical cancer. *Br J Cancer* **97**: 1135-1138

Nosho K, Baba Y, Tanaka N, Shima K, Hayashi M, Meyerhardt JA, Giovannucci E, Dranoff G, Fuchs CS, Ogino S (2010) Tumour-infiltrating T-cell subsets, molecular changes in colorectal cancer, and prognosis: Cohort study and literature review. *J Pathol* **222**: 350-366

Perrone G, Ruffini PA, Catalano V, Spino C, Santini D, Muretto P, Spoto C, Zingaretti C, Sisti V, Alessandroni P, Giordani P, Cicetti A, D'Emidio S, Morini S, Ruzzo A, Magnani M, Tonini G, Rabitti C, Graziano F (2008) Intratumoural FOXP3-positive regulatory T cells are associated with adverse prognosis in radically resected gastric cancer. *Eur J Cancer* **44**: 1875-1882

Prall F, Duhrkop T, Weirich V, Ostwald C, Lenz P, Nizze H, Barten M (2004) Prognostic role of CD8+ tumor-infiltrating lymphocytes in stage III colorectal cancer with and without microsatellite instability. *Hum Pathol* **35**: 808-816

Ruffini E, Asioli S, Filosso PL, Lyberis P, Bruna MC, Macri L, Daniele L, Oliaro A (2009) Clinical significance of tumor-infiltrating lymphocytes in lung neoplasms. *Ann Thorac Surg* **87**: 365-371

Sasaki A, Tanaka F, Mimori K, Inoue H, Kai S, Shibata K, Ohta M, Kitano S, Mori M (2008) Prognostic value of tumor-infiltrating FOXP3+ regulatory T cells in patients with hepatocellular carcinoma. *Eur J Surg Oncol* **34**: 173-179

Sato E, Olson SH, Ahn J, Bundy B, Nishikawa H, Qian F, Jungbluth AA, Frosina D, Gnjatic S, Ambrosone C, Kepner J, Odunsi T, Ritter G, Lele S, Chen YT, Ohtani H, Old LJ, Odunsi K (2005) Intraepithelial CD8+ tumor-infiltrating lymphocytes and a high CD8+/regulatory T cell ratio are associated with favorable prognosis in ovarian cancer. *Proc Natl Acad Sci U S A* **102**: 18538-18543

Shah CA, Allison KH, Garcia RL, Gray HJ, Goff BA, Swisher EM (2008) Intratumoral T cells, tumor-associated macrophages, and regulatory T cells: association with p53 mutations, circulating tumor DNA and survival in women with ovarian cancer. *Gynecol Oncol* **109**: 215-219

Shen Z, Zhou S, Wang Y, Li RL, Zhong C, Liang C, Sun Y (2010) Higher intratumoral infiltrated Foxp3+ Treg numbers and Foxp3+/CD8+ ratio are associated with adverse prognosis in resectable gastric cancer. *J Cancer Res Clin Oncol* **136**: 1585-1595

Shimizu K, Nakata M, Hirami Y, Yukawa T, Maeda A, Tanemoto K (2010) Tumor-infiltrating Foxp3+ regulatory T cells are correlated with cyclooxygenase-2 expression and are associated with recurrence in resected non-small cell lung cancer. *J Thorac Oncol* **5**: 585-590

Siddiqui SA, Frigola X, Bonne-Annee S, Mercader M, Kuntz SM, Krambeck AE, Sengupta S, Dong H, Cheville JC, Lohse CM, Krco CJ, Webster WS, Leibovich BC, Blute ML, Knutson KL, Kwon ED (2007) Tumor-infiltrating Foxp3-CD4+CD25+ T cells predict poor survival in renal cell carcinoma. *Clin Cancer Res* **13**: 2075-2081

Simpson JA, Al Attar A, Watson NF, Scholefield JH, Ilyas M, Durrant LG (2010) Intratumoral T cell infiltration, MHC class I and STAT1 as biomarkers of good prognosis in colorectal cancer. *Gut* **59**: 926-933

Sinicrope FA, Rego RL, Ansell SM, Knutson KL, Foster NR, Sargent DJ (2009) Intraepithelial effector (CD3+)/regulatory (FoxP3+) T-cell ratio predicts a clinical outcome of human colon carcinoma. *Gastroenterology* **137**: 1270-1279

Sorbye SW, Kilvaer T, Valkov A, Donnem T, Smeland E, Al Shibli K, Bremnes RM, Busund LT (2011) Prognostic impact of lymphocytes in soft tissue sarcomas. *PLoS One* **6**: e14611

Stumpf M, Hasenburg A, Riener MO, Jutting U, Wang C, Shen Y, Orlowska-Volk M, Fisch P, Wang Z, Gitsch G, Werner M, Lassmann S (2009) Intraepithelial CD8-positive T lymphocytes predict survival for patients with serous stage III ovarian carcinomas: relevance of clonal selection of T lymphocytes. *Br J Cancer* **101**: 1513-1521

Tierney JF, Stewart LA, Ghersi D, Burdett S, Sydes MR (2007) Practical methods for incorporating summary time-to-event data into meta-analysis. *Trials* **8**: 16

Tomsova M, Melichar B, Sedlakova I, Steiner I (2007) Prognostic significance of CD3+ tumor-infiltrating lymphocytes in ovarian carcinoma. *Gynecol Oncol*

Toomey D, Smyth G, Condron C, Kelly J, Byrne AM, Kay E, Conroy RM, Broe P, Bouchier-Hayes D (2003) Infiltrating immune cells, but not tumour cells, express FasL in non-small cell lung cancer: No association with prognosis identified in 3-year follow-up. *Int J Cancer* **103**: 408-412

Wakabayashi O, Yamazaki K, Oizumi S, Hommura F, Kinoshita I, Ogura S, Dosaka-Akita H, Nishimura M (2003) CD4+ T cells in cancer stroma, not CD8+ T cells in cancer cell nests, are associated with favorable prognosis in human non-small cell lung cancers. *Cancer Sci* **94**: 1003-1009

Zhang L, Conejo-Garcia JR, Katsaros D, Gimotty PA, Massobrio M, Regnani G, Makrigiannakis A, Gray H, Schlienger K, Liebman MN, Rubin SC, Coukos G (2003) Intratumoral T cells, recurrence, and survival in epithelial ovarian cancer. *N Engl J Med* **348**: 203-213

Zingg U, Montani M, Frey DM, Dirnhofer S, Esterman AJ, Went P, Oertli D (2010) Tumour-infiltrating lymphocytes and survival in patients with adenocarcinoma of the oesophagus. *Eur J Surg Oncol* **36**: 670-677

Zlobec I, Lugli A, Baker K, Roth S, Minoo P, Hayashi S, Terracciano L, Jass JR (2007) Role of APAF-1, E-cadherin and peritumoral lymphocytic infiltration in tumour budding in colorectal cancer. *J Pathol* **212**: 260-268
